# TNF-Mediated Inhibition of Classical Swine Fever Virus Replication Is IRF1-, NF-κB- and JAK/STAT Signaling-Dependent

**DOI:** 10.3390/v13102017

**Published:** 2021-10-07

**Authors:** Matthias Liniger, Markus Gerber, Sandra Renzullo, Obdulio García-Nicolás, Nicolas Ruggli

**Affiliations:** 1 Institute of Virology and Immunology IVI, Sensemattstrasse 293, 3147 Mittelhäusern, Switzerland; matthias.liniger@ivi.admin.ch (M.L.); markus.gerber@ivi.admin.ch (M.G.); sandra.renzullo@ivi.admin.ch (S.R.); obdulio.garcia-nicolas@ivi.admin.ch (O.G.-N.); 2 Department of Infectious Diseases and Pathobiology (DIP), Vetsuisse Faculty, University of Bern, Länggassstrasse 122, 3012 Bern, Switzerland

**Keywords:** pestivirus, classical swine fever virus, CSFV, TNF, type I IFN, JAK/STAT, NF-κB, IRF1, IRF3, IFNAR1, CRISPR/Cas9

## Abstract

The sera from pigs infected with virulent classical swine fever virus (CSFV) contain substantial amounts of tumor necrosis factor (TNF), a prototype proinflammatory cytokine with pleiotropic activities. TNF limits the replication of CSFV in cell culture. In order to investigate the signaling involved in the antiviral activity of TNF, we employed small-molecule inhibitors to interfere specifically with JAK/STAT and NF-κB signaling pathways in near-to-primary endothelial PEDSV.15 cells. In addition, we knocked out selected factors of the interferon (IFN) induction and signaling pathways using CRISPR/Cas9. We found that the anti-CSFV effect of TNF was sensitive to JAK/STAT inhibitors, suggesting that TNF induces IFN signaling. Accordingly, we observed that the antiviral effect of TNF was dependent on intact type I IFN signaling as PEDSV.15 cells with the disrupted type I IFN receptor lost their capacity to limit the replication of CSFV after TNF treatment. Consequently, we examined whether TNF activates the type I IFN induction pathway. With genetically modified PEDSV.15 cells deficient in functional interferon regulatory factor 1 or 3 (IRF1 or IRF3), we observed that the anti-CSFV activity exhibited by TNF was dependent on IRF1, whereas IRF3 was dispensable. This was distinct from the lipopolysaccharide (LPS)-driven antiviral effect that relied on both IRF1 and IRF3. In agreement with the requirement of IRF1 to induce TNF- and LPS-mediated antiviral effects, intact IRF1 was also essential for TNF- and LPS-mediated induction of IFN-β mRNA, while the activation of NF-κB was not dependent on IRF1. Nevertheless, NF-κB activation was essential for the TNF-mediated antiviral effect. Finally, we observed that CSFV failed to counteract the TNF-mediated induction of the IFN-β mRNA in PEDSV.15 cells, suggesting that CSFV does not interfere with IRF1-dependent signaling. In summary, we report that the proinflammatory cytokine TNF limits the replication of CSFV in PEDSV.15 cells by specific induction of an IRF1-dependent antiviral type I IFN response.

## 1. Introduction

The first line of protection of host cells from invading viruses is mediated by the innate immune system. By sensing unique pathogen-associated molecular patterns, conserved cellular pattern recognition receptors initiate multiple intracellular signaling cascades involving interferon regulatory factors (IRF) that culminate in the transcriptional activation and secretion of type I interferons (IFN-α and IFN-β) and type III IFN (IFN-λ) [1]. Specific interactions of IFNs with cellular type I (IFNAR1) and type III IFN receptors subsequently activate Janus kinase (JAK)- and signal transducer and activator of transcription (STAT)-dependent signaling in neighboring cells. Consequently, the IFN-mediated JAK/STAT signaling leads to the expression of a multitude of IFN-stimulated genes that synergistically orchestrate cellular antiviral defense [2].

Classical swine fever virus (CSFV) causes a highly contagious hemorrhagic fever in pigs [3]. CSFV is a non-cytopathogenic pestivirus of the Flaviviridae family, and the enveloped virion harbors a single-stranded positive-sense RNA genome [4]. Like most viruses, CSFV is highly susceptible to the antiviral actions mediated by type I and type III IFN and has evolved potent strategies to interfere with the cellular antiviral defense [3,5,6,7]. In most cells except plasmacytoid dendritic cells (pDC), CSFV interferes with type I IFN induction by means of the viral N^pro^ protein which interacts with IRF3 and induces its proteasomal degradation [7,8]. IRF3 is a key transcription factor of the type I IFN induction cascade triggered by DNA and RNA viruses that is targeted by many viral and bacterial pathogens [9]. Despite N^pro^-mediated IRF3 degradation, CSFV induces potent IFN-α and proinflammatory host responses in vivo involving pDC, conventional DC and monocytic cells (reviewed in [7]). Among the proinflammatory cytokines, tumor necrosis factor (TNF) represents a key cytokine promoting pleiotropic cellular effects, such as apoptosis, proliferation, survival or differentiation [10]. TNF activates nuclear factor κB (NF-κB) and mitogen-activated protein kinase signaling pathways [11]. Notably, pigs infected with virulent CSFV induce high levels of TNF [12,13,14], and TNF was reported to inhibit the replication of CSFV in porcine cells [15,16]. The antiviral effect of TNF was reduced in p65-silenced PK-15 cells indicating that TNF inhibits CSFV replication via the NF-κB signaling pathway [16]. Interestingly, porcine reproductive and respiratory syndrome virus (PRRSV) infection leads to TNF secretion that in turn inhibits the proliferation of a subsequent CSFV C-strain infection, which may explain CSFV vaccination failures caused by PRRSV infection in the field [15].

Studies conducted with primary macrophages and murine microvascular endothelial cells revealed that TNF induces IRF1-dependent IFN-β responses [17,18,19]. Like IRF3, IRF1 does also bind and activate the IFN-β promoter [20]. Furthermore, IRF1 is critical for the TNF-driven type I IFN response in rheumatoid fibroblast-like synoviocytes [21]. Interestingly, CSFV infection or dsRNA stimulation of PK-15 cells upregulate IRF1 mRNA [22,23]. Overexpression of IRF1 in PK-15 cells triggers antiviral responses against different porcine viruses, although IRF1 is dispensable for IFN-β induction by RNA viruses [23]. Finally, a recent study showed that CSFV N^pro^ antagonizes IRF1-mediated type III IFN production by downregulating IRF1 expression and inhibiting its nuclear translocation in a porcine intestinal epithelial cell line [24].

Altogether, the data described above show that antiviral TNF signaling involves NF-κB and IRF1 and that the anti-CSFV activity of TNF relies on type I IFN responses in an IRF1- and/or IRF3-dependent manner, but the formal proof for a direct link of these signaling elements in the context of CSFV is still missing. In order to explore this in more details, we aimed at deciphering the cellular signaling pathways exhibited by TNF-driven anti-CSFV responses using pharmacological and genetic targeting of selected cellular signaling factors. For this, we used the immortalized near-to-primary porcine aortic endothelial cell line PEDSV.15 [25] that we found to be highly sensitive to the antiviral action triggered by physiological levels of TNF, including porcine (pTNF) and murine TNF (mTNF), as opposed to the common porcine kidney cell lines PK-15 and SK-6. For quantitative virological readouts, we employed a firefly luciferase-expressing CSFV (CSFV-luc). With inhibitory drugs, CRISPR/Cas9-mediated gene knockout and anti-TNF antibodies, we demonstrate that TNF limits the replication of CSFV by activating JAK/STAT signaling in an IRF1-, NF-κB and IFNAR1-dependent way, independently of IRF3.

## 2. Materials and Methods

### 2.1. Cells

The immortalized porcine aortic endothelial cell line PEDSV.15 [25] was maintained in Dulbecco’s modified Eagle medium (DMEM, Thermo Fisher Scientific, Waltham, MA, USA) containing sodium pyruvate, nonessential amino acids (NEAA), 7% horse serum (SVA, Hatunaholm, Sweden) and 2% porcine serum (Gibco, Thermo Fisher Scientific, Waltham, MA, USA). The porcine kidney cell lines PK-15 and SK-6 were propagated respectively in DMEM and in Earle’s minimal essential medium (MEM, Thermo Fisher Scientific, Waltham, MA, USA), each supplemented with 7% horse serum. Monocyte-derived macrophages (MDM) were isolated from blood from specific pathogen-free Large White pigs bred at the IVI, Mittelhäusern, Switzerland, essentially as described earlier [26]. Blood collection was performed in compliance with the animal welfare regulations of Switzerland under the cantonal licenses BE131/17 and BE127/2020. Peripheral blood mononuclear cells were collected using density gradient centrifugation (1.077 g/liter; Ficoll-Paque Plus, Cytiva, Marlborough, MA, USA). Monocytes (CD172a^+^ cells) were then isolated by magnetic cell sorting using LS columns (Miltenyi Biotec, Bergisch Gladbach, Germany) and the monoclonal antibody 74-22-15A (HB-142.1, ATCC, Manassas, VA, USA). Sorted monocytes were seeded at 5 × 10^5^ cells/mL in DMEM supplemented with 10% pestivirus-free fetal bovine serum and porcine macrophage colony-stimulating factor (20 U/mL, produced at the IVI [26]) and cultured for 3 days at 39 °C and 5% CO_2_ for differentiation to MDM.

### 2.2. Viruses

The bicistronic CSFV-luc was derived from a full-length cDNA construct obtained by replacing the N^pro^-C gene cassette in the pA187-1 cDNA backbone [27] with the corresponding N^pro^-Luc-IRES-C gene cassette from the bicistronic pA187-N^pro^-Luc-IRES-C-delE^rns^ replicon construct [28] using standard PCR-mediated cloning. The CSFV-luc and the virulent CSFV vEy-37 [29] were rescued from cDNA as described elsewhere [30] and propagated in PEDSV.15 cells. Viral titers were determined by endpoint dilution in PEDSV.15 cells and expressed as 50% tissue culture infectious dose (TCID_50_)/mL. CSFV E2 was detected in infected cell monolayers by immunoperoxidase staining with the HC/TC-26 monoclonal anti-E2 hybridoma supernatant [31] as described elsewhere [32].

### 2.3. Reagents

Recombinant porcine TNF-alpha was purchased from R&D Systems (Minneapolis, MN, USA, cat. No. 690-PT-025) whereas murine TNF was produced in-house [33]. The TLR3 ligand polyinosinic:polycytidylic acid (p(I:C)) and the *E. coli*-derived TLR4 ligand LPS were purchased from Sigma-Aldrich (St. Louis, MO, USA). TPCA-1 and ruxolitinib were purchased from Selleckchem (Houston, TX, USA). The alamarBlue cell viability reagent was obtained from Thermo Fisher Scientific (Waltham, MA, USA). Adalimumab (Humira, Abbott Laboratories, Chicago, IL, USA), a human recombinant IgG1 monoclonal antibody that neutralizes human TNF, was purchased from Selleckchem (Houston, TX, USA).

### 2.4. Antiviral TNF Assay and JAK/STAT Compound Library Screening

The PEDSV.15 cells seeded in 96-well plates (3 × 10^4^ cells/100 µL/well) were treated with small-molecule compounds of the JAK/STAT Compound Library (Targetmol, Wellesley Hills, MA, USA, cat. No. L3700) at two concentrations (0.5 µM and 5 µM) for approximately one hour prior to stimulation with either LPS (100 ng/mL), pTNF (5 ng/mL) or the medium. After a stimulation period of six hours, the cells were infected with CSFV-luc at a multiplicity of infection (MOI) of 0.1 TCID_50_/cell, and after 22 h of cultivation, the cell extracts were assayed for firefly luciferase activity (Firefly Luciferase Assay Kit 2.0, Biotium, Fremont, CA, USA) using a Centro LB 960 luminometer (Berthold Technologies, Bad Wildbad, Germany). Average relative luminescence units (RLU) with standard deviations from triplicate values were calculated. The data obtained from cytotoxic or antiviral compounds were eliminated from the analysis (RLU below 50% of non-stimulated infected cultures).

### 2.5. RNA Isolation and Quantification

For real-time RT-PCR, RNA was extracted from porcine cells grown in six-well plates using a NucleoSpin RNA kit (Macherey-Nagel, Düren, Germany) and quantified with AgPath-ID One-Step RT-PCR reagents using an ABI PRISM 7700 sequence detector system (Applied Biosystems, Thermo Fisher Scientific, Waltham, MA, USA). The relative expression of each mRNA was determined with the ΔCt method by calculating the amount of target mRNA in relation to 18S mRNA. The following oligonucleotides and probes purchased from Microsynth (Balgach, Switzerland) were used: 18S forward primer 5′-CGC CGC TAG AGG TGA AAT TC-3′; 18S reverse primer 5′-GGC AAA TGC TTT CGC TCT G-3′; 18S probe 5′-TGG ACC GGC GCA AGA CGG A-3′; IFN-β forward primer 5′-GGC TGG AAT GAA ACC GTC AT-3′; IFN-β reverse primer 5′-TCC AGG ATT GTC TCC AGG TCA-3′; IFN-β probe 5′-CCT TGT GGA ACT TGA TGGGCA GAT GG-3′ [34].

### 2.6. NF-κB Promoter Reporter Assay

The NF-κB reporter plasmid, pGL4.32[luc2P/NF-κB-RE/Hygro] (Promega, Madison, WI, USA), contains five copies of an NF-κB response element (NF-κB-RE) that drives the transcription of the firefly luciferase reporter gene. Briefly, 3 million PEDSV.15 cells in 0.4 mL ice-cold PBS were electroporated with 5 µg pGL4.32[luc2P/NF-κB-RE/Hygro] and 200 ng pGL4.75[hRluc/CMV] vector (Promega, Madison, WI, USA) reporter plasmids. The cells were seeded in 96-well plates (3 × 10^4^ cells/100 µL/well), and after overnight incubation, the cells were treated with inhibitors or DMSO for 30 min and stimulated for six hours. Cell extracts were prepared in 25 μL of 1× passive lysis buffer per well (Biotium, Fremont, CA, USA). The samples were assayed for firefly and *Renilla* luciferase activities using the Firefly & Renilla Luciferase Single Tube Assay Kit (Biotium, Fremont, CA, USA) and a Centro LB 960 luminometer (Berthold Technologies, Bad Wildbad, Germany).

### 2.7. Generation of IFNAR1, IRF3 and IRF1 Gene Knockout PEDSV.15 Cell Lines Using CRISPR/Cas9 Gene Editing

Based on the publicly available mRNA sequences for porcine IFNAR1 (NCBI reference NM_213772.1), IRF3 (NM_213770.1) and IRF1 (XM_021080244.1) individual guide RNAs (gRNAs) for the targeting of corresponding genomic exons were designed. CRISPR/Cas9-based genome editing was performed essentially as described [35]. The two *Bbs*I restriction endonuclease sites of the plasmid pSpCas9(BB)-2A-GFP (PX458) (Plasmid #48138, Addgene, Watertown, MA, USA) were used to clone annealed oligonucleotides coding for the selected gRNAs. Individual gRNAs were designed using the CHOPCHOP web tool (http://chopchop.cbu.uib.no/, accessed on 30 November 2020) [36]. The gRNA target sequences with protospacer adjacent motifs (PAM) are shown in Table 1 and oligonucleotides for annealing and subsequent cloning are shown in Table 2. The PEDSV.15 cells seeded in six-well plates were cotransfected with a pair of pSpCas9(BB)-2A-GFP (PX458)-derivative plasmids encompassing separate gRNAs to create short genomic deletions in early exons using Lipofectamine 2000 (Thermo Fisher Scientific, Waltham, MA, USA). IRF1 was only targeted with a single pSpCas9(BB)-2A-GFP (PX458)-derivative construct. GFP-positive cells were sorted two days after the transfection using fluorescence-activated cell sorting (FACSAria, Becton Dickinson, Franklin Lakes, NJ, USA). Individual cell clones were obtained after limiting dilution and clonal expansion. The cell clones were screened by PCR-based methods. Briefly, genomic DNA was extracted from cells using a commercial kit (NucleoSpin DNA RapidLyse, Macherey-Nagel, Düren, Germany) and selected genomic loci were amplified by PCR using a Phusion Hot Start II DNA Polymerase (Thermo Fisher Scientific, Waltham, MA, USA) and specific pairs of oligonucleotides (Microsynth, Balgach, Switzerland) (Table 3). The amplicons were cloned in pCR4-TOPO and verified by DNA sequencing using an ABI 3130 Genetic Analyzer (Thermo Fisher Scientific, Waltham, MA, USA).

### 2.8. Western Blot Analyses

The cells were lysed with a denaturing lysis buffer composed of 62.5 mM Tris HCl (pH 6.8), 2% sodium dodecyl sulfate (SDS), 10% glycerol and 0.05% bromophenol blue. The proteins were separated using 4–12% gradient SDS–polyacrylamide gel electrophoresis under nonreducing conditions (ExpressPlus, GenScript, Piscataway, NJ, USA) and analyzed by means of Western blotting using PVDF transfer membranes (Immobilon-FL, Merck Millipore, Burlington, MA, USA) and an Odyssey Infrared Imaging System (LI-COR Biosciences, Bad Homburg, Germany). Porcine IRF3 and viral N^pro^ proteins were detected using the rabbit anti-IRF3 and anti-N^pro^ sera as described previously [8,37]. Using the mouse monoclonal Anti-β-Actin Antibody C4 (Santa Cruz Biotechnology, Dallas, TX, USA), β-actin was detected as the loading control.

## 3. Results

### 3.1. TNF Inhibits CSFV Replication in Porcine PEDSV.15 Cells and MDM, but Not in the PK-15 and SK-6 Cell Lines

**Figure 1:**
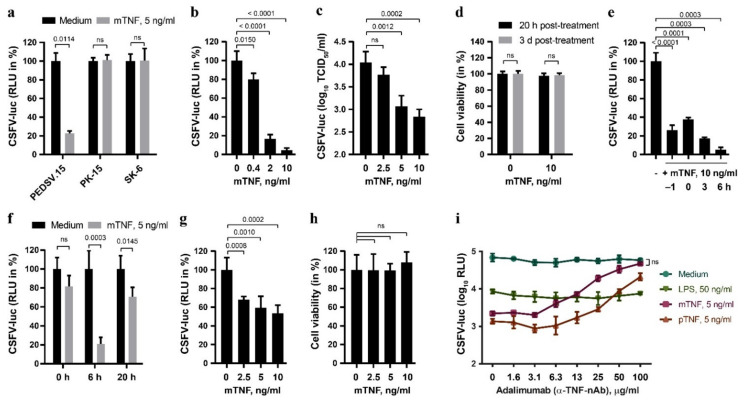
TNF antagonizes CSFV infection in porcine aortic endothelial cells and in primary porcine MDM, but not in PK-15 and SK-6 cells. (**a**) The PEDSV.15, PK-15 and SK-6 cells were treated with mTNF (5 ng/mL) or the medium for five hours. After the washing step with the medium, the cells were infected with CSFV-luc at a MOI of 0.1 TCID_50_/cell. The firefly luciferase activity in cell culture lysates was determined 20 h after infection. (**b**,**c**) Similarly, the PEDSV.15 cells were treated with increasing concentrations of mTNF prior to CSFV-luc infection and quantification of luciferase activity from cell lysates (**b**) or viral titers from cell culture supernatants (**c**) 20 h later. (**d**) The parallel mock- (0) and mTNF-treated CSFV-luc-infected PEDSV.15 cell cultures were analyzed for cell viability using the alamarBlue cell viability assay at the indicated times and the values are shown as percentage of untreated cells. (**e**) The antiviral effect of mTNF in the PEDSV.15 cells was assessed in time-of-addition experiments. (**f**) The pretreated cells were infected and cultured in the presence of mTNF until termination of the experiments 20 h after infection. (**g**) Porcine MDM were stimulated for six hours with increasing concentrations of mTNF prior to CSFV-luc infection (MOI 0.2 TCID_50_/cell) and measurement of luciferase activity in cell culture lysates 20 h later. (**h**) Viability of the TNF-treated and infected MDM was assessed in parallel as in (**d**). (**i**) The PEDSV.15 cells were preincubated for six hours with mTNF, pTNF and LPS in the presence of increasing concentrations of adalimumab, a human TNF-neutralizing antibody, prior to CSFV-luc replication analysis as described above. In panels (**a**,**b**,**e**–**g**), the firefly luciferase activity is shown as the percentage of the medium control in the absence of TNF. The data represent the mean of three (**i**), four (**a**–**c**,**e**,**f**), five (**g**,**h**) or 12 (**d**) experimental replicas with error bars indicating the standard deviations. Statistically significant differences (*p* < 0.05) were determined using the unpaired, two-tailed Student’s *t*-test (ns, nonsignificant).

CSFV-infected pigs show elevated serum TNF, and TNF was shown to inhibit replication in cell lines [15,16]. In order to characterize the antiviral activity of TNF against CSFV more extensively, we quantified the effect of TNF of different origin on the replication of CSFV expressing a firefly luciferase reporter (CSFV-luc) in primary porcine cells versus permanent cell lines (Figure 1). The near-to-primary endothelial cell line PEDSV.15 [25] responded to mTNF with a significant reduction of CSFV-mediated luciferase activity 20 h after infection, which was not observed in the PK-15 and SK-6 cells, two permanent porcine cell lines used commonly to propagate CSFV (Figure 1a). The PEDSV.15 cells stimulated for six hours with increasing concentrations of mTNF, from 0.4 ng/mL to 10 ng/mL, displayed a dose-dependent reduction of CSFV replication, as determined by CSFV-mediated luciferase activity (Figure 1b) and by titration of infectious viruses from cell culture supernatants (Figure 1c). Notably, the TNF treatment did not affect the viability of PEDSV.15 cells at 20 h or three days post-treatment (Figure 1d). Time-of-addition experiments revealed the highest mTNF-mediated inhibition of CSFV infection after six hours of treatment (Figure 1e). Prolonged overnight TNF treatment of the PEDSV.15 cells did not result in an enhanced antiviral state (Figure 1f). TNF pre-stimulation of MDM did also interfere with CSFV (Figure 1g), although not as strongly as in the PEDSV.15 cells (Figure 1b), without affecting the viability of the cells (Figure 1h). This suggests differences in TNF responsiveness of MDM versus PEDSV.15 cells. In order to examine the specificity of mTNF and pTNF for triggering the antiviral effects observed, we employed adalimumab (Humira, Abbott Laboratories), a neutralizing human anti-hTNF monoclonal antibody [38]. TNF treatment in presence of increasing concentrations of adalimumab reduced specifically the antiviral effect of TNF but not of lipopolysaccharide (LPS), known to induce a TLR4-dependent antiviral type I IFN response (Figure 1i). This was observed with both mTNF and pTNF. Notably, mTNF neutralization blocked the antiviral TNF activity completely and specifically. Altogether, these data demonstrate that TNF inhibits CSFV replication in primary porcine cells but not in PK-15 and SK-6.

### 3.2. The Anti-CSFV Activity of TNF Involves JAK/STAT Signaling

**Figure 2:**
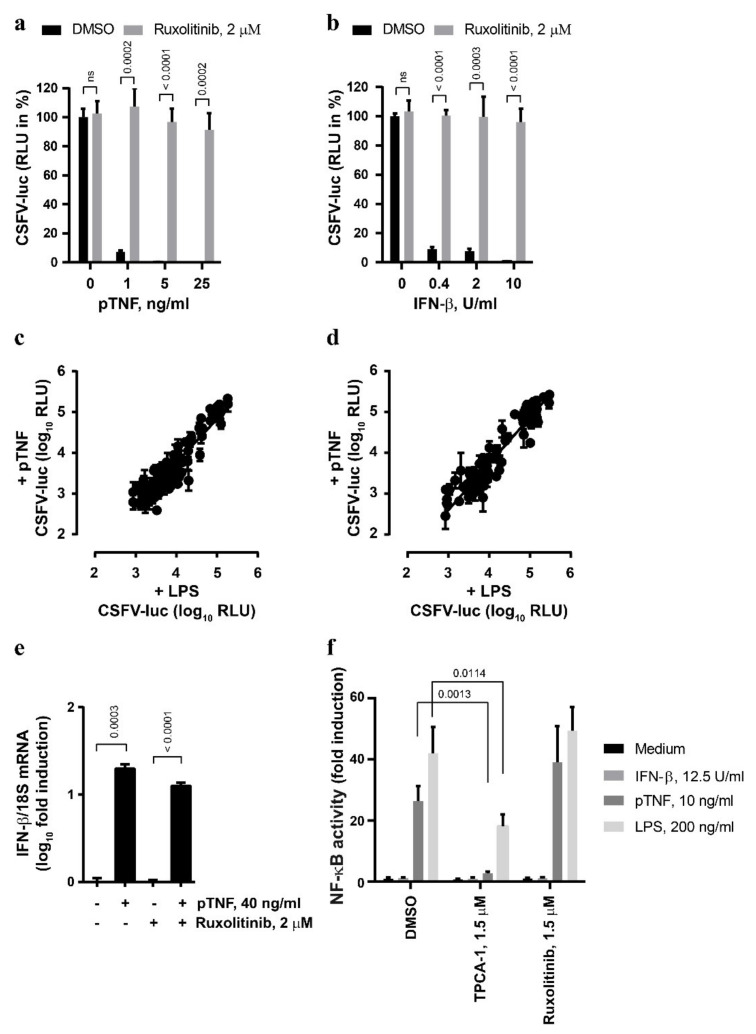
The anti-CSFV activity of TNF involves JAK/STAT signaling. (**a**,**b**) The PEDSV.15 cells were treated with pTNF (**a**) or IFN-β (**b**) in the presence of ruxolitinib (2 µM) or DMSO for six hours prior to CSFV-luc infection (MOI, 0.1 TCID_50_/cell) and measurement of firefly luciferase activity 22 h later. The values are shown as the percentage of the DMSO control in the absence of pTNF or IFN-β, respectively. (**c**,**d**) JAK/STAT inhibitor Compound Library screens were performed with CSFV-luc-infected cells pre-stimulated with the medium, LPS or pTNF in the presence of the individual JAK/STAT inhibitors at the concentration of 0.5 µM (**c**) or 5 µM (**d**). The data due to cytotoxic and antiviral activity of the compounds were eliminated from the analysis. (**e**) The PEDSV.15 cells were treated with pTNF or mock in the presence or absence of ruxolitinib for four hours, and the IFN-β mRNA to 18S ribosomal RNA ratio was quantified by means of RT-qPCR and plotted as log_10_ fold induction. (**f**) The PEDSV.15 cells were transfected with an NF-κB-dependent firefly luciferase gene reporter plasmid and treated with IFN-β, pTNF, LPS in the presence of the NF-κB signaling inhibitor TPCA-1 or the JAK/STAT inhibitor ruxolitinib. The NF-κB activity is represented as fold luciferase induction related to the medium treatment. The data represent the means and the standard deviations of three independent experimental replicas. The differences were considered to be statistically significant at *p* < 0.05 using the Student’s *t*-test (ns, nonsignificant).

The JAK/STAT pathway is the key element of the signaling cascade engaged in response to type I IFN [39]. In order to explore whether this pathway is also involved in the antiviral action of TNF, we targeted JAK/STAT signaling with small-molecule inhibitors. Strikingly, the antiviral effects of pTNF and mTNF were sensitive to the JAK inhibitor ruxolitinib (Figure 2a and Supplementary Figure S1a, respectively) that also blunted the antiviral IFN-β signaling as expected (Figure 2b). Under these conditions, neither ruxolitinib treatment nor mTNF stimulation affected the viability of the PEDSV.15 cells (Supplementary Figure S1b). In order to screen for potential unique features of the classical LPS- and pTNF-mediated antiviral signaling in the context of CSFV infection, we employed a JAK/STAT Compound Library (see Materials and Methods) composed of a collection of 145 compounds targeting JAKs (JAK1, JAK2, JAK3 and TYK2) and STATs (STAT1, STAT2, STAT3, STAT4, STAT5A, STAT5B and STAT6).

Figure 2c,d depicts plotted log_10_ RLU values from CSFV-luc-infected cells pre-stimulated with LPS or pTNF in the presence of the individual JAK/STAT inhibitors at concentrations of 0.5 µM or 5 µM, respectively. Altogether, we observed a general and strong correlation between the capacities of the compounds to interfere with LPS- and pTNF-mediated antiviral signaling without any outliers that would reduce LPS activity without affecting pTNF activity. This suggests that pTNF induces classical antiviral JAK/STAT signaling in PEDSV.15 cells. The effect of selected well-characterized JAK/STAT inhibitors, such as fedratinib, itacitinib, neratinib, pacritinib, tofacitinib, filgotinib, baricitinib and ruxolitinib efficiently blocked both LPS- and pTNF-mediated antiviral effects, which is shown in Supplementary Figure S1c,d for drug concentrations of 0.5 µM or 5 µM, respectively. JAK/STAT signaling is triggered typically by type I IFNs. Therefore, we assessed whether TNF induces the IFN-β mRNA in PEDSV.15 cells. As expected, elevated IFN-β mRNA levels were detected four hours after pTNF stimulation (Figure 2e). The pTNF-mediated upregulation of the IFN-β mRNA was independent of the JAK inhibitor ruxolitinib, suggesting that pTNF elicits a direct induction of the IFN-β promoter. Despite several attempts, we failed to detect bioactive type I interferon in cell culture supernatants of LPS- and pTNF-stimulated PEDSV.15 cells using a firefly luciferase-based MX-promoter assay or a sensitive VSV-luc-based assay. By applying a transient firefly luciferase reporter gene assay for NF-κB-dependent promoter activity (NF-κB-RE), we observed JAK/STAT-independent activation of NF-κB with pTNF- and LPS-stimulated PEDSV.15 cells (Figure 2f). As expected, IFN-β stimulation did not induce the NF-κB response element. TNF-mediated NF-κB activation was sensitive to TPCA-1, a selective small-molecule inhibitor of IκB kinase 2 known to inhibit NF-κB nuclear localization. Ruxolitinib, on the contrary, did not inhibit the pTNF- and LPS-mediated NF-κB-dependent promoter activation, indicating that this activation was JAK/STAT-independent. The discrepancy between the JAK/STAT-dependent antiviral activity and the JAK/STAT-independent activation of an NF-κB-dependent promoter suggests that pTNF-mediated induction of NF-κB-dependent pathways is not sufficient to trigger the antiviral effect. In conclusion, we observed that in porcine PEDSV.15 cells, pTNF stimulates a JAK/STAT-specific antiviral response, induces IFN-β mRNA and activates JAK/STAT-independent NF-κB signaling.

### 3.3. The Anti-CSFV Activity of TNF Requires the Type I IFN Receptor, While IRF3 Is Dispensable

**Figure 3:**
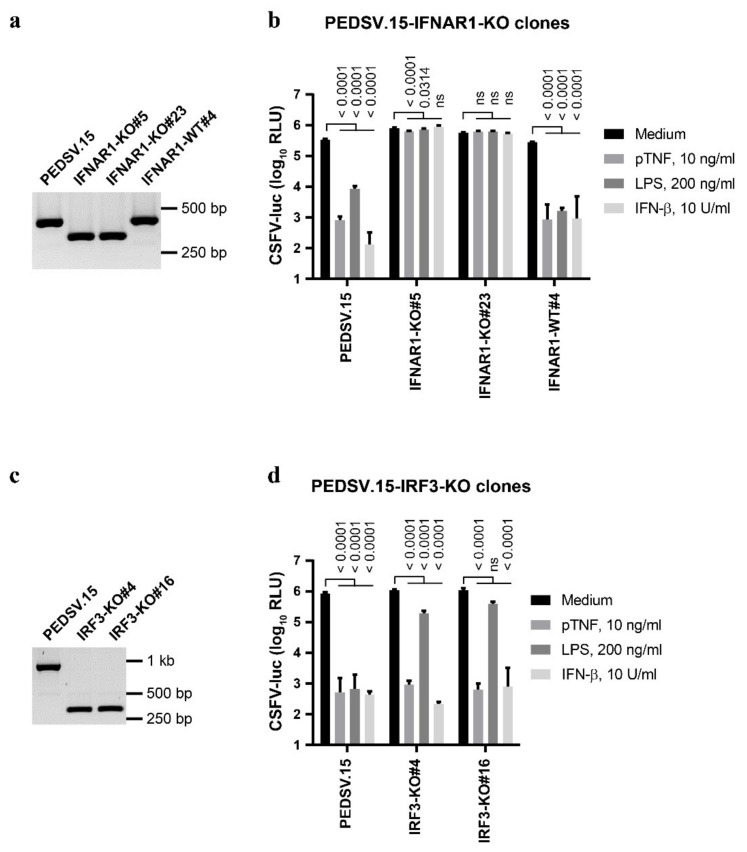
The anti-CSFV activity of TNF requires IFNAR1, while IRF3 is dispensable. PEDSV.15 cells lacking functional IFNAR1 or IRF3 were generated using CRISPR/Cas9-based genome editing by targeting early exons with two separate gRNAs each. (**a**,**c**) PCR amplifications of the IFNAR1 (**a**) and IRF3 (**c**) loci are shown for the parent cells and for two knockout cell clones each. A Cas9-exposed clone with a functional receptor (IFNAR1-WT#4) served as the negative control. (**b**,**d**) The IFNAR1- (**b**) or IRF3-KO cells (**d**) were stimulated with pTNF, LPS, IFN-β or the medium for seven hours followed by infection with CSFV-luc at a MOI of 0.1 TCID_50_/cell for 22 h before cell lysates were processed for firefly luciferase measurement. The data in (**b**,**d**) represent the means and the standard deviations of six independent experimental replicas. Significant differences compared with the medium (*p* < 0.05) were calculated with one-way ANOVA and post hoc tests (the *p*-values are indicated; ns, nonsignificant).

In order to explore the roles of the type I IFN receptor and of IRF3 in antiviral IFN-β, LPS and pTNF signaling, we generated IFNAR1- and IRF3-knockout (KO) PEDSV.15 cell lines (IFNAR1-KO and IRF3-KO, respectively) by introducing small genetic deletions within early exons using CRISPR/Cas9 gene editing (Figure 3). We determined the respective genotypes after editing and clonal expansion using PCR combined with Sanger DNA sequencing.

We identified two IFNAR1-KO clones #5 and #23 carrying deletions within the IFNAR1 loci (Figure 3a) consisting of heterozygous open reading frame disruptions. One allele from each clone encodes an mRNA with an internal deletion after the first 72 codons leading to a frameshift mutation. The other alleles have an in-frame deletion leading to a 39 amino acid (aa) deletion after aa position 72. This resulted in functional disruption of IFNAR1, since the two clones (#5 and #23) lost the capacity to establish antiviral states upon IFN-β, LPS and pTNF stimulation, contrary to the parent PEDSV.15 cells (Figure 3b). One PEDSV.15 clone with two intact wild-type (WT) IFNAR1 loci called IFNAR1-WT#4 served as the Cas9-exposed negative control and responded to all three stimuli similar to the parent PEDSV.15 cells (Figure 3b). Collectively, these data confirm that IFNAR1 is necessary for the antiviral activity triggered by IFN-β and LPS and demonstrate the requirement of the type I IFN receptor for the antiviral signaling induced by pTNF.

For IRF3, we identified three knockout PEDSV.15 clones with identical out-of-frame homozygous deletions of 190 nucleotides within the IRF3 open reading frame on the deduced mRNA level, leading to a frameshift mutation after the first 38 codons. The IRF3-KO clones #4 and #16 (Figure 3c) served for functional analyses (Figure 3d). During isolation of IRF3-KO clones, we did not obtain any unedited Cas9-exposed negative control. However, since we performed the stimulations in parallel with the IRNAR1-KO clones, the unedited IFNAR1-WT#4 cells (Figure 3b) served as the Cas9-exposed negative control for the IRF3-KO cells. As expected, IRF3-KO cells maintained their capacity to respond to IFNAR1-dependent IFN-β stimulation (Figure 3d). Importantly, the disruption of IRF3 resulted in a fundamental difference between LPS- and pTNF-triggered antiviral innate immune responses. While the LPS-mediated antiviral state was mostly abolished in IRF3-KO cells, IRF3 was completely dispensable for the pTNF-mediated anti-CSFV activity (Figure 3d). This highlights the mechanistic differences in the initiation of innate immune responses between pTNF- and LPS-triggered signaling.

### 3.4. The Anti-CSFV Activities of LPS and TNF Are IRF1-Dependent

**Figure 4:**
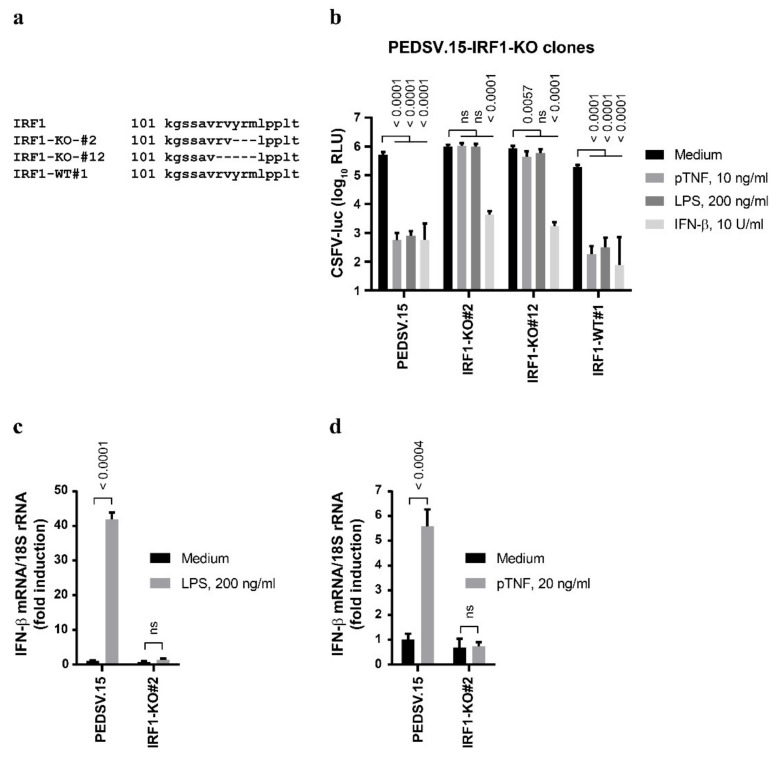
The anti-CSFV activities of LPS and TNF are IRF1-dependent. (**a**) PEDSV.15 cells expressing nonfunctional IRF1 were generated with CRISPR/Cas9-based genome editing. (**b**) Two independent knockout clones (#2 and #12) and the Cas9-exposed negative control (IRF1-WT#1) with intact IRF1 were stimulated with pTNF, LPS, IFN-β or the medium for seven hours followed by infection with CSFV-luc at a MOI of 0.1 TCID_50_/cell for 22 h before the cell lysates were processed for firefly luciferase measurement. (**c**,**d**) The effect of two hours of stimulations with LPS (**c**) or pTNF (**d**) on the expression of IFN-β mRNA normalized to 18S ribosomal RNA was assessed in the PEDSV.15 and IRF1-KO#2 cells. The data in (**b**) represent the means and the standard deviations of six independent experimental replicas and significant differences compared with the medium (*p* < 0.05) were calculated with one-way ANOVA and post hoc tests (*p*-value indicated; ns, nonsignificant). The data in (**c**,**d**) represent the means and the standard deviations of three independent experimental replicas. Significant differences compared with the medium (*p* < 0.05) were calculated with the unpaired, two-tailed Student’s *t*-test (the *p*-values are indicated; ns, nonsignificant).

Besides IRF3, IRF1 can also trigger type I IFN induction [20]. Therefore, we generated functional IRF1-KO PEDSV.15 cells using CRISPR/Cas9 to test whether pTNF-mediated antiviral responses require IRF1. We obtained two IRF1-KO clones—IRF1-KO#2 and IRF1-KO#12—that carried homozygous genomic deletions without disruption of the deduced IRF1 open reading frames at the mRNA level (deletions relative to the ORF nucleotide positions 322–331, TGTACCGGA and nucleotide positions 316–331, TGCGGGTGTACCGGA, respectively). This resulted in mutant IRF1 proteins harboring the small deletions ΔYRM (aa positions 109–111) and ΔRVYRM (aa positions 107–111) for the IRF1-KO#2 and IRF1-KO#12 clones, respectively. These deletions are located within the C-terminal region of the IRF tryptophan pentad repeat DNA-binding domain (aa positions 5–113, https://prosite.expasy.org/, accessed on 2 July 2021) (Figure 4a). The clone IRF1-WT#1 with intact IRF1 loci served as the Cas9-exposed control cell line.

Similar to the parent PEDSV.15 and the IRF3-KO cells (see Figure 3d), the IRF1-KO cells responded normally to IFN-β, resulting in the inhibition of CSFV replication through an intact type I IFN signaling pathway (Figure 4b). However, unlike the PEDSV.15 and the IRF1-WT#1 cells, the IRF1-KO cell clones #2 and #12 were unable to respond to antiviral doses of LPS and pTNF, demonstrating that in PEDSV.15 cells, IRF1 possesses essential functions to mediate both LPS- and pTNF-driven antiviral effects. Interestingly, the disruption of LPS- and pTNF-mediated antiviral signaling in IRF1-KO cells coincides with impaired induction of the IFN-β mRNA. Compared with the PEDSV.15 cells, we observed impaired IFN-β mRNA upregulation in the IRF1-KO#2 cells two hours after LPS (Figure 4c) and pTNF stimulation (Figure 4d), demonstrating the essential function of IRF1 in LPS- and pTNF-mediated induction type I IFN.

### 3.5. The Anti-CSFV Activity of TNF Is NF-κB-Dependent, but NF-κB Can Function Independently of IRF1

**Figure 5:**
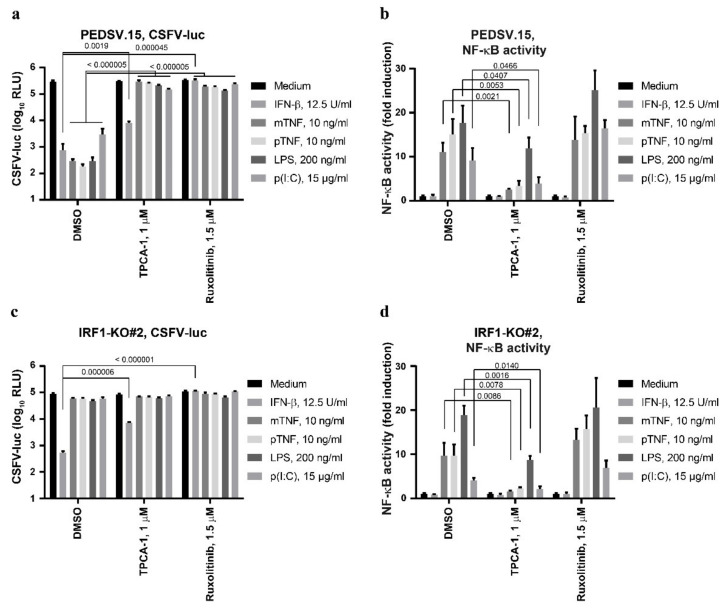
The anti-CSFV activity of TNF depends on NF-κB, but NF-κB can function independently of IRF1. The PEDSV.15 (**a**,**b**) or PEDSV.15-derived IRF1-KO cells (IRF1-KO#2) (**c**,**d**) were transfected with the NF-κB reporter plasmid 18 h prior to stimulation (**b**,**d**) or left untransfected (**a**,**c**) and stimulated with IFN-β, mTNF, pTNF, LPS or p(I:C) in the absence (DMSO) or presence of TPCA-1 or ruxolitinib at the indicated concentrations. The untransfected cells (**a**, **c**) were infected with CSFV-luc seven hours after stimulation. Firefly luciferase (**a**,**c**) or firefly and *Renilla* dual-luciferase activities (**b**,**d**) were measured 24 h after infection or six hours after stimulation, respectively. The NF-κB-dependent promoter activity is plotted as fold induction compared to the medium. The data represent the means and the standard deviations of at least three independent experimental replicas. Significant differences compared with the medium (*p* < 0.05) were calculated with the unpaired, two-tailed Student’s *t*-test (the *p*-values are indicated; ns, nonsignificant).

The induction of type I IFN depends on NF-κB, as opposed to the downstream IFN-β signaling [40]. Therefore, we tested the role of NF-κB in mTNF- and pTNF-mediated anti-CSFV activity using the NF-κB inhibitor TPCA-1 and included LPS, p(I:C) and IFN-β as control stimulations (Figure 5a). TPCA-1 prevented the antiviral actions of mTNF, pTNF, LPS and p(I:C), but not of IFN-β. These results confirm that NF-κB signaling is required for the induction of antiviral activity; however, it is dispensable for downstream IFN-β signaling. Accordingly, the JAK inhibitor ruxolitinib efficiently blocked mTNF, pTNF, LPS, p(I:C) and IFN-β-driven antiviral effects as expected. In parallel, we measured the activation of NF-κB after stimulation of the PEDSV.15 cells (Figure 5b). Treatment with mTNF-, pTNF-, LPS- and p(I:C), but not with IFN-β, resulted in NF-κB activation, which was JAK/STAT-independent (see also Figure 2f). In contrast, mTNF-, pTNF-, LPS- and p(I:C)-mediated NF-κB activation was sensitive to TPCA-1, demonstrating the specificity of the drug. As demonstrated previously, IRF1-KO cells were unable to induce antiviral actions triggered by TNF and LPS (Figure 4b and Figure 5c), which was also the case for the p(I:C) trigger (Figure 5c). Interestingly, despite impaired induction of an antiviral state in IRF1-KO cells, we noted intact mTNF-, pTNF-, LPS- and p(I:C)-mediated NF-κB responses, implying that IRF1 is not involved in the activation of NF-κB-dependent signaling (Figure 5d). In summary, we observed that TNF-, LPS- and p(I:C)-mediated activation of NF-κB is required for the establishment of antiviral activity against CSFV, but that NF-κB-dependent signals can function independently of IRF1.

### 3.6. CSFV Infection Does Not Interfere with TNF- and LPS-Mediated IFN-β mRNA Induction in PEDSV.15 Cells

**Figure 6:**
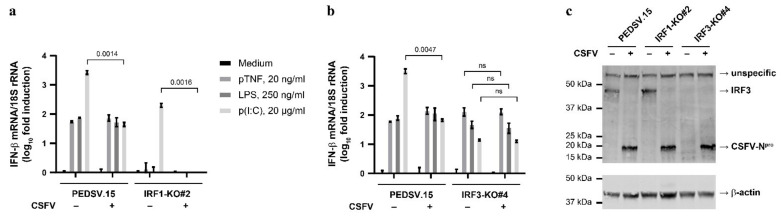
CSFV infection does not interfere with TNF- or LPS-mediated induction of IFN-β mRNA in PEDSV.15 cells. The PEDSV.15 (**a**,**b**), IRF1-KO#2 (**a**) and IRF3-KO#4 cells (**b**) were mock-infected (–) or infected with CSFV vEy-37 (+) for 3 days and stimulated subsequently for 2.5 h with pTNF, LPS or p(I:C) at the indicated concentrations or left untreated (medium). After stimulation, IFN-β mRNA induction normalized with 18S RNA was quantified by RT-qPCR. (**c**) Parallel cultures of the mock- (–) or CSFV-infected (+) PEDSV.15, IRF1-KO#2 and IRF3-KO#4 cells were lysed and the proteins were separated by SDS–PAGE and analyzed by means of Western blotting for IRF3, CSFV N^pro^ and β-actin expression. The data in (**a**,**b**) show the means and the standard deviations of three independent experimental replicas. Statistically significant differences were determined with the unpaired, two-tailed Student’s *t*-test (the *p*-values are indicated; ns, nonsignificant).

CSFV antagonizes the induction of type I IFN by means of N^pro^ through IRF3 targeting [8]. In addition, a recent report showed that N^pro^ inhibits the expression and nuclear translocation of IRF1, thereby suppressing the production of type III IFN [24]. Therefore, we hypothesized that CSFV may antagonize the TNF-induced IRF1-dependent and the LPS-induced IRF1-/IRF3-dependent IFN-β mRNA induction in PEDSV.15 cells. In order to address this, we infected the PEDSV.15, IRF1-KO#2 and IRF3-KO#4 cells with the virulent CSFV strain vEy-37 for 3 days prior to stimulation with pTNF, LPS or p(I:C) and measured the induction of IFN-β mRNA in comparison with the stimulated mock-infected cells (Figure 6a,b). Mock- and CSFV-infected PEDSV.15 cells had comparable IFN-β mRNA levels after pTNF or LPS stimulation (Figure 6a,b). This was different with p(I:C), where pre-infected PEDSV.15 cells had significantly lower levels of IFN-β mRNA than mock-infected cells. Similarly, the p(I:C)-mediated induction of IFN-β mRNA was sensitive to CSFV in IRF1-KO#2 cells (Figure 6a). As expected (see Figure 4c,d), the IRF1-KO#2 cells did not respond with IFN-β mRNA to pTNF or LPS stimulation (Figure 6a). With IRF3-KO#4 cells (Figure 6b), CSFV pre-infection had no significant effect on the pTNF, LPS and p(I:C)-mediated induction of IFN-β mRNA, suggesting that CSFV is unable to interfere with IRF1-dependent antiviral signaling. At the time of LPS or pTNF treatment, all the infected cells were positive for the virus antigen, as shown by immunostaining of the E2 protein (Supplementary Figure S2). Unfortunately, we were unable to detect endogenous IRF1 protein by Western blot analysis. However, as expected from our previous studies with PK-15 cells [8], the IRF3 protein was degraded in the CSFV-infected PEDSV.15 and IRF1-KO#2 cells (Figure 6c), which is consistent with reduced or absent IFN-β mRNA induction upon p(I:C) stimulation (Figure 6a,b). Notably, IRF3 was not detected in IRF3-KO#4 cells, confirming successful genome editing leading to IRF3 protein knockout. Collectively, these findings indicate that CSFV lacks countermeasures to interfere with IRF1-dependent TNF- and LPS-mediated type I IFN induction in PEDSV.15 cells.

## 4. Discussion

The induction of high levels of proinflammatory cytokines including TNF is a hallmark of severe and hemorrhagic CSF following infection with highly pathogenic CSFV [7]. Several independent studies report secretion of TNF peaking at 100–500 pg/mL serum 4–5 days after infection of pigs with CSFV [12,13,14]. CSFV-infected alveolar macrophages can also secrete up to 1 ng/mL TNF at 16 h after infection [41]. TNF was reported to inhibit CSFV replication in porcine PK-15 cells [16] and may be at the origin of the vaccination failure with live-attenuated CSFV in PRRSV-infected pigs [15]. Therefore, this study aimed at dissecting the intracellular signaling cascade required for the anti-CSFV activity of TNF.

First, we observed that different cell types responded differently to TNF. While TNF induced an antiviral state in the endothelial cell line PEDSV.15 and in the porcine MDM, TNF did not inhibit CSFV replication in the SK-6 and PK-15 cells (Figure 1a). This was unexpected given the TNF-mediated inhibition of CSFV replication in PK-15 cells reported earlier [16]. Differences in the steady-state levels of rate-limiting factors such as IRF1 may explain this discrepancy. A different degree of dedifferentiation in general may be a reason for the difference between the PEDSV.15 and MDM cultures and the permanent cell lines used commonly for the propagation of CSFV. The PEDSV.15 are immortalized porcine aortic endothelial cells that maintained most morphological and functional properties of primary endothelial cells and were therefore proposed to serve as a prototypical alternative to normal endothelial cells [25]. The MDM were primary cells prepared from porcine blood (see materials and methods). These results emphasize the importance of cell type-dependent differences in cellular responses to infection. Further investigation may include selected approaches such as comparison of the IRF1 levels and activity, as well as high-throughput differential transcriptomic and proteomic analyses.

Next, we dissected the TNF–IRF1–IFN-β signaling axis in the context of a CSFV infection of PEDSV.15 cells. More specifically, we showed that TNF, similarly to LPS, induces the expression of IFN-β transcripts by activating IRF1 and NF-κB independently. Thereby, TNF triggers type I IFN receptor-dependent JAK/STAT signaling leading to decreased CSFV replication. Synergistic antiviral effects between TNF and IFNs are known to enhance antiviral responses (reviewed in [18]). Although we cannot rule out such cooperative effects with TNF stimulations, we were able to efficiently blunt the antiviral effects of mTNF and pTNF with an anti-TNF neutralizing antibody (Figure 1i), which confirms the specific activity exhibited by the TNF formulations. The antiviral effects mediated by TNF and LPS were similarly sensitive to individual compounds of a JAK/STAT inhibitor library (Figure 2c,d). Importantly, the downstream antiviral effects of TNF were independent of the type III IFN antiviral pathway in the PEDSV.15 cells since there was a strict IFNAR1 requirement (Figure 3b). This may be different in other cell types such as intestinal epithelial cells that induce type III IFNs in an IRF1-dependent manner [24]. In the PEDSV.15 cells, we demonstrated that the TNF-triggered induction of IFN-β transcripts and the resulting antiviral effect rely on intact IRF1 (Figure 4b,d), whereas IRF3 was dispensable (Figure 3d). IRF1 is also required for adequate LPS-mediated induction of IFN-β mRNA and subsequent antiviral activity (Figure 4b,c). However, in contrast to TNF that solely depends on IRF1, LPS also requires functional IRF3 to establish its antiviral effect. The latter is consistent with the well-established MyD88-independent LPS/TLR4 signal transduction pathway (reviewed in [42]). Previous studies conducted with primary macrophages, murine microvascular endothelial cells and rheumatoid fibroblast-like synoviocytes described TNF-mediated IRF1-dependent type I IFN responses [17,19,21], but this study shows for the first time inhibition of CSFV replication through this axis.

Besides IRF1, NF-κB is also required for the TNF-mediated antiviral effect on CSFV (Figure 5). Li et al. showed that TNF interferes with CSFV replication via the NF-κB signaling pathway as the antiviral TNF effect was lost in p65-silenced PK-15 cells [16]. Our observation that the antiviral effect of TNF is strictly IRF1- and IFNAR1-dependent (Figure 3 and Figure 4) and that NF-κB activation is independent of functional IRF1 (Figure 5) demonstrates clearly that the induction of the type I IFN pathway is required besides NF-κB activation for interference with CSFV replication.

Interestingly, CSFV did not interfere with TNF- or LPS-triggered IFN-β mRNA induction (Figure 6) that both depend on IRF1 (Figure 4c,d). However, as expected from our previous studies with PK-15 cells [8], CSFV prevented p(I:C)-mediated IFN-β mRNA induction in the PEDSV.15 and IRF1-KO#2 cells, which was consistent with the absence of IRF3 when N^pro^ was expressed (Figure 6). These results are not surprising when considering that CSFV targets specifically IRF3 for proteasomal degradation by means of N^pro^ [8] while the TNF-induced anti-CSFV activity we observed in the PEDSV.15 cells was completely independent of IRF3 (Figure 3d). However, the lack of interference of CSFV with the IRF1-dependent pathway described here is in apparent contradiction with recent data showing that N^pro^ of CSFV inhibits IRF1 expression and nuclear translocation in porcine intestinal epithelial IPEC-J2 cells, thereby suppressing type III IFN production [24]. This latter finding may, however, be a specific feature of IFN-λ producing cells. The fact that TNF exhibits substantial anti-CSFV activity in certain cell types and that TNF is secreted in response to CSFV infection in pigs may imply that CSFV evolved yet unidentified means of antagonizing antiviral signaling triggered by TNF in vivo. This is matter of ongoing and future studies.

## 5. Conclusions

Several reports suggest altogether that the antiviral activity of TNF involves NF-κB- and IRF1-dependent signaling and type I IFN responses. In order to test this formally for CSFV, we targeted NF-κB, IRF1, IRF3 and IFNAR1-dependent JAK/STAT signaling pharmacologically or by CRISPR/Cas9-mediated gene knockout. The anti-CSFV activity of porcine and murine TNF was inhibited by antibody-mediated TNF neutralization, NF-κB and JAK/STAT inhibitors and was abrogated completely in the IRF1 and IFNAR gene knockout cells but not in the IRF3 gene knockout cells. IRF1 gene knockout prevented TNF- and LPS-mediated IFN-β mRNA induction. Interestingly, CSFV did not counteract TNF- or LPS-mediated IFN-β mRNA induction. This is consistent with CSFV targeting IRF3 for proteasomal degradation [8] but is in apparent contradiction with CSFV-mediated inhibition of IRF1-dependent signaling reported recently [24]. The latter may be restricted to specific cell types that induce type III IFN. Whether CSFV does selectively inhibit the antiviral activity of TNF through IRF1 targeting in mucosal cells still needs to be explored. Nevertheless, the findings of this study contribute to a better understanding of the CSF immunopathogenesis and of the virus–host interaction of CSFV. More generally, this knowledge is valuable for the development of antiviral and immunoprophylactic interventions.

## Figures and Tables

**Table 1:** Target sequences (gRNA).

Target	Target Sequence (gRNA) ^1^
IFNAR1-target1	GATAATTGGATAAAGTTGCCTGG
IFNAR-target2	CAGGAAACAGCACTTCTCCGTGG
IRF3-target1	GCCGCAAGCCGTGCTTCCAAGGG
IRF3-target2	TAGATCTTGTGTGGGTCGTGGGG
IRF1-target2	GCTCAGCTGTGCGGGTGTACCGG

^1^ The PAM sequences are underlined.

**Table 2:** Oligonucleotides for annealing and cloning into *Bbs*I of plasmid pSpCas9(BB)-2A-GFP (PX458).

Oligonucleotide	Sequence (5′-3′) ^1^
IFNAR1_CC9_1F	caccgGATAATTGGATAAAGTTGCC
IFNAR1_CC9_1R	aaacGGCAACTTTATCCAATTATCc
IFNAR1_CC9_2F	caccgCAGGAAACAGCACTTCTCCG
IFNAR1_CC9_2R	aaacCGGAGAAGTGCTGTTTCCTGc
IRF3_CC9_1F	caccgGCCGCAAGCCGTGCTTCCAA
IRF3_CC9_1R	aaacTTGGAAGCACGGCTTGCGGCc
IRF3_CC9_2F	caccgTAGATCTTGTGTGGGTCGTG
IRF3_CC9_2R	aaacCACGACCCACACAAGATCTAc
IRF1_CC9_2F	caccgGCTCAGCTGTGCGGGTGTAC
IRF1_CC9_2R	aaacGTACACCCGCACAGCTGAGCc

^1^ The gRNA target sequences are capitalized.

**Table 3:** Oligonucleotides for the amplification of edited genomic regions.

Oligonucleotide	Sequence (5′-3′) (gRNA)
IFNAR1-gF	TTGGTATGTGTGCATTGAAAGA
IFNAR1-gR	ATGAGCTTGGGAAGTGAACTGT
IRF3-gF	CTGATATCTCAGCTGAACCAGG
IRF3-gR2	GGTATCAGAGGTACTGTATC
IRF1-gF	TGTGTATAGGCAGGCATACGAG
IRF1-gR	ACTGAGGCTTGCTGGATGTATT

## Data Availability

All the data are included in the manuscript.

## Supplementary Materials

The following are available online at https://www.mdpi.com/article/10.3390/v13102017/s1, Figure S1: The anti-CSFV activity of TNF involves JAK/STAT signaling, Figure S2: Verification of CSFV infection of PEDSV.15, IRF1-KO#2 and IRF3-KO#4 cells from the experiments in Figure 6.
