# Supplementary material for: TNF-Mediated Inhibition of Classical Swine Fever Virus Replication Is IRF1-, NF-κB- and JAK/STAT Signaling-Dependent
Source: Viruses. 2021 Oct 7;13(10):2017. doi: 10.3390/v13102017 (PMC8540133; doi:10.3390/v13102017)
Supplement: Supplementary file 1 [file viruses-13-02017-s001.zip › viruses-1330461-supplementary.pdf]

Article

TNF-Mediated Inhibition of Classical Swine Fever Virus Replication Is IRF1-, NF-κB- and JAK/STAT Signaling-Dependent

Matthias Liniger [1,2], Markus Gerber [1,2], Sandra Renzullo [1], Obdulio García-Nicolás [1,2] and Nicolas Ruggli [1,2,*]

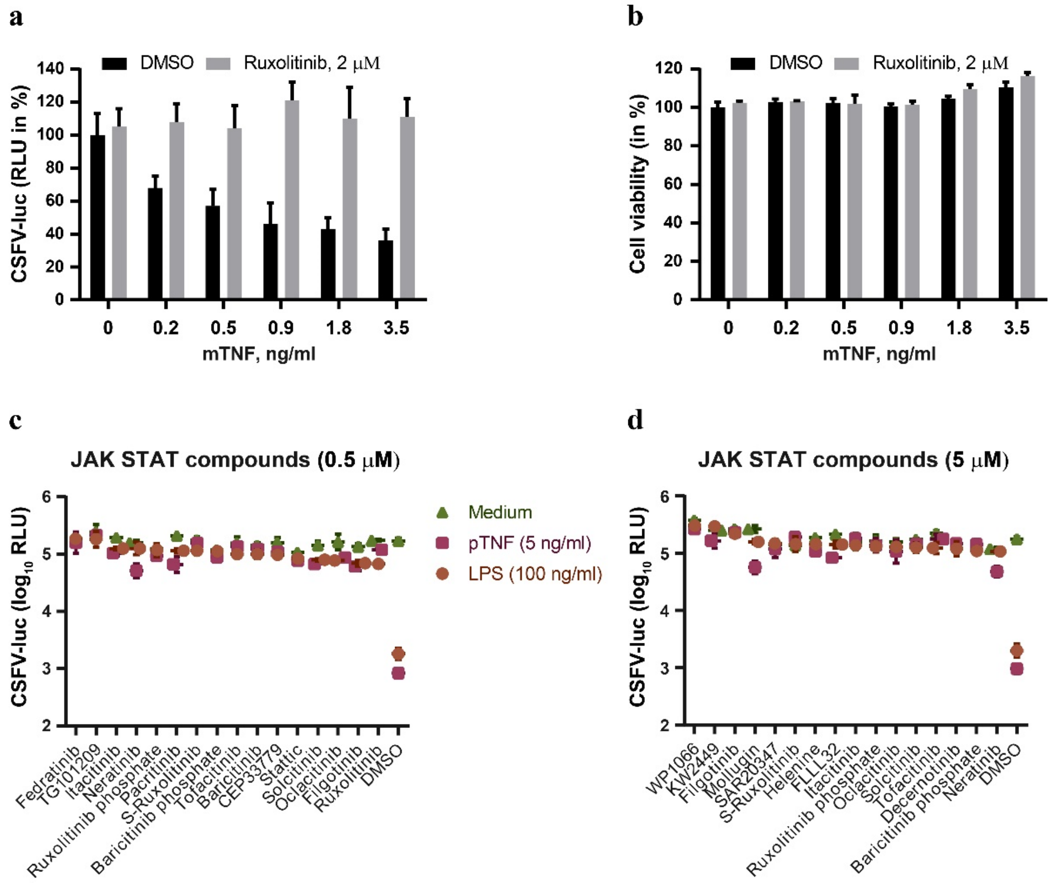

Figure S1. The anti-CSFV activity of TNF involves JAK/STAT signaling. (**a**) The effect of the treatments of PEDSV.15 cells with increasing concentrations of mTNF in presence or absence of ruxolitinib on the replication of CSFV-luc is shown. (**b**) In parallel, the cells were assayed for cell viability. (**c**,**d**) Selected JAK/STAT inhibitors that blocked LPS and pTNF-mediated antiviral effects are depicted individually, for drug concentrations of 0.5 μM (**c**) or 5 μM (**d**), respectively.

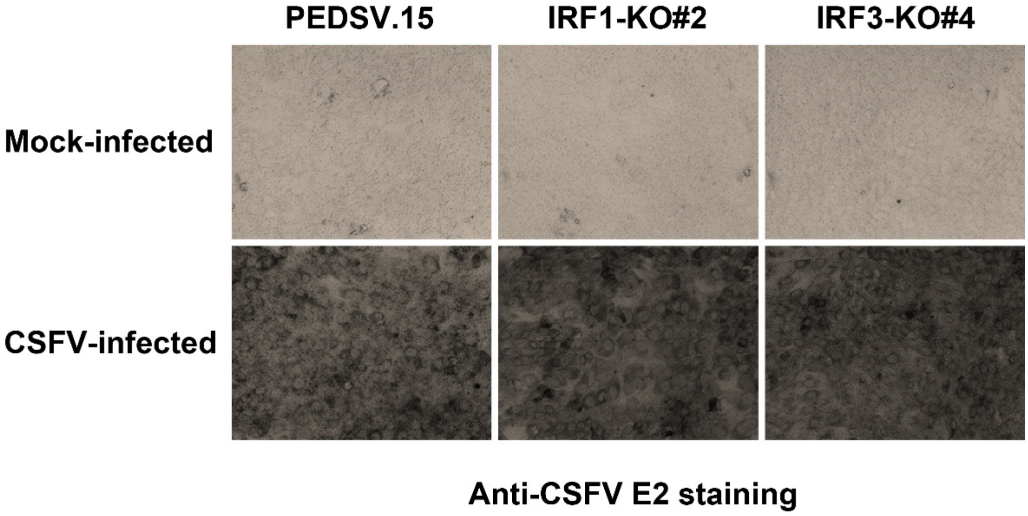

Figure S2. Verification of CSFV infection of PEDSV.15, IRF1-KO#2 and IRF3-KO#4 cells from the experiments of Figure 6. Mock- and CSFV vEy-37-infected cells were analyzed 3 days post-infection by immunoperoxidase staining for viral E2 using the HC/TC-26 anti-E2 monoclonal antibody.
